# Area-Selective Atomic Layer Deposition of ZnO on Si\SiO_2_ Modified with Tris(dimethylamino)methylsilane

**DOI:** 10.3390/ma16134688

**Published:** 2023-06-29

**Authors:** Behnam Moeini, Tahereh G. Avval, Hidde H. Brongersma, Stanislav Průša, Pavel Bábík, Elena Vaníčková, Brian R. Strohmeier, David S. Bell, Dennis Eggett, Steven M. George, Matthew R. Linford

**Affiliations:** 1 Department of Chemistry and Biochemistry, Brigham Young University, Provo, UT 84602, USA; behnam.moeini@gmail.com (B.M.); tgholianavval@gmail.com (T.G.A.); 2 Department of Applied Physics, Eindhoven University of Technology, 5600 MB Eindhoven, The Netherlands; h.h.brongersma@tue.nl; 3 Institute of Physical Engineering, Brno University of Technology, Technická 2, 616 69 Brno, Czech Republic; prusa@fme.vutbr.cz (S.P.); pavel.babik@ceitec.vutbr.cz (P.B.); 187500@vutbr.cz (E.V.); 4 CEITEC BUT, Brno University of Technology, Purkyňova 123, 612 00 Brno, Czech Republic; 5 Materials Group-NA, Avery Dennison Corporation, 8080 Norton Parkway, Mentor, OH 44060, USA; brian.strohmeier@averydennison.com; 6 Restek Corporation, 110 Benner Circle, Bellefonte, PA 16823, USA; david.bell@restek.com; 7 Department of Statistics, Brigham Young University, Provo, UT 84602, USA; dennis_eggett@byu.edu; 8 Department of Chemistry, University of Colorado, 215 UCB, Boulder, CO 80309, USA; steven.george@colorado.edu

**Keywords:** area selective, atomic layer deposition, silane, silicon, inhibitor, ZnO

## Abstract

Delayed atomic layer deposition (ALD) of ZnO, i.e., area selective (AS)-ALD, was successfully achieved on silicon wafers (Si\SiO_2_) terminated with tris(dimethylamino)methylsilane (TDMAMS). This resist molecule was deposited in a home-built, near-atmospheric pressure, flow-through, gas-phase reactor. TDMAMS had previously been shown to react with Si\SiO_2_ in a single cycle/reaction and to drastically reduce the number of silanols that remain at the surface. ZnO was deposited in a commercial ALD system using dimethylzinc (DMZ) as the zinc precursor and H_2_O as the coreactant. Deposition of TDMAMS was confirmed by spectroscopic ellipsometry (SE), X-ray photoelectron spectroscopy (XPS), and wetting. ALD of ZnO, including its selectivity on TDMAMS-terminated Si\SiO_2_ (Si\SiO_2_\TDMAMS), was confirmed by in situ multi-wavelength ellipsometry, ex situ SE, XPS, and/or high-sensitivity/low-energy ion scattering (HS-LEIS). The thermal stability of the TDMAMS resist layer, which is an important parameter for AS-ALD, was investigated by heating Si\SiO_2_\TDMAMS in air and nitrogen at 330 °C. ALD of ZnO takes place more readily on Si\SiO_2_\TDMAMS heated in the air than in N_2_, suggesting greater damage to the surface heated in the air. To better understand the in situ ALD of ZnO on Si\SiO_2_\TDMAMS and modified (thermally stressed) forms of it, the ellipsometry results were plotted as the normalized growth per cycle. Even one short pulse of TDMAMS effectively passivates Si\SiO_2_. TDMAMS can be a useful, small-molecule inhibitor of ALD of ZnO on Si\SiO_2_ surfaces.

## 1. Introduction

Atomic layer deposition (ALD) is a powerful, advanced, thin-film deposition technique that is based on self-terminating surface reactions [1,2]. It meets the need for atomic layer control/deposition of materials in many scientific fields, facilitating advances in fabrication processes and methods. In its most common embodiment, ALD involves two self-terminating reactions that are used in an alternating fashion to (ideally) deposit up to a monolayer of a material on a surface with each ALD cycle. Between the two ALD half reactions, unreacted reagent is pumped/purged away. When the half reactions are well chosen and properly implemented, ALD allows precise control over film thickness and uniformity. ALD is an example of bottom-up thin-film fabrication because it is literally based on the atom-by-atom growth of thin films. ALD can uniformly coat patterned/irregular surfaces that are difficult to coat by other techniques; an important advantage of ALD is the conformality of the films that are often created.

Area-selective ALD (AS-ALD) is an important focus of current research in ALD [3,4]. AS-ALD has also been referred to as substrate-dependent deposition [3]. In AS-ALD, surfaces are modified to inhibit or direct subsequent ALD growth [3,4]. Thus, surface chemistry plays a central role in AS-ALD [5,6], and differences in surface chemistry can control feature sizes. There are two main approaches to AS-ALD: activation or passivation [7]. However, while surface activation is a viable approach to AS-ALD, surface passivation is its core focus because it is cheaper and more scalable [8]. There are also other AS-ALD methods such as selective precursor and co-reactant adsorption methods [9,10]. Two general approaches for inhibiting a reaction at a surface are (i) to prevent a reactant’s diffusion to the active functional groups on a surface via self-assembled monolayers, [11] and (ii) to passivate/end-cap these groups with small inhibitor molecules [7,12]. The first approach is based on sterics and the second on chemical derivatization. Although both approaches are appropriate for AS-ALD, small inhibitor/end-capping molecules often enjoy greater success because they are often both volatile and their deposition compatible with current industrial tools and processes [8].

**Figure 1:**
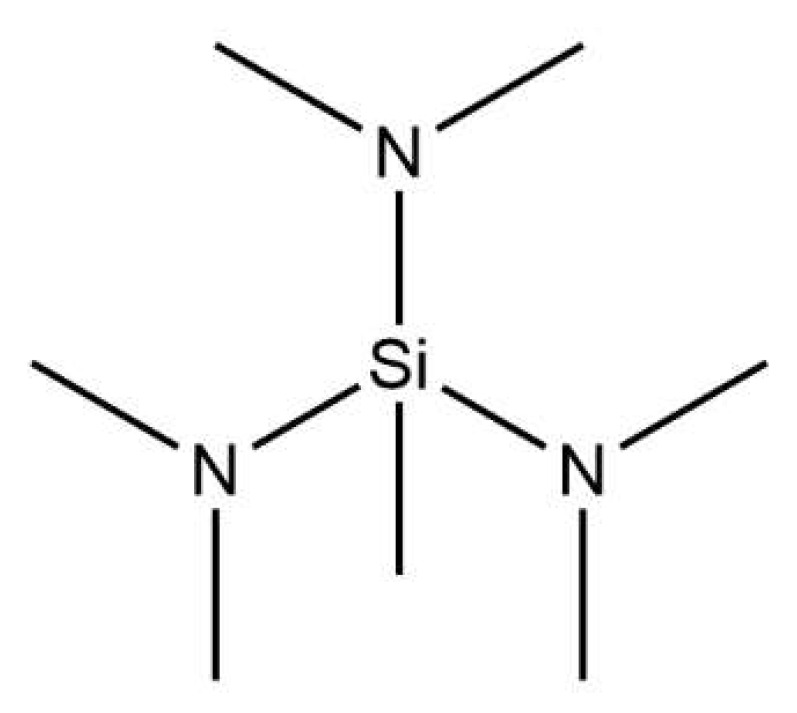
Structure of tris(dimethylamino)methylsilane (TDMAMS).

Recently, we demonstrated the surface passivation of the inner surfaces of silica capillary columns for gas chromatography using a small silane (tris(dimethylamino)methylsilane, TDMAMS), (see Figure 1), delivered by a near atmospheric pressure gas-phase reactor [13]. This deposition was also followed on witness silicon shards. The reaction of surface silanols with TDMAMS was confirmed by X-ray photoelectron spectroscopy (XPS), spectroscopic ellipsometry (SE), contact angle goniometry (wetting), and high-sensitivity/low-energy ion scattering (HS-LEIS). HS-LEIS showed that the reaction of Si\SiO_2_ with TDMAMS results in a large drop in the number of free surface silanols (from 3.66 to 0.31 silanols/nm^2^ on native-oxide-terminated silicon). This very large decrease in the surface silanol concentration caused us to ask whether TDMAMS-terminated silicon oxide (Si\SiO_2_\TDMAMS) might inhibit ALD. Accordingly, in this work, we investigate the ALD of ZnO on Si\SiO_2_ and Si\SiO_2_\TDMAMS, where TDMAMS was again deposited in the home-built, flow-through, atmospheric pressure, gas-phase reactor [14]. ZnO is an important metal oxide with applications in optoelectronics, sensing, memory devices, catalysis, transparent conducting oxides, and thin-film transistors [15,16,17,18,19]. In addition, its properties can be controlled by doping with elements such as indium (In) and gallium (Ga) [20,21]. In our work, ZnO was deposited by thermal ALD of dimethylzinc (DMZ) and H_2_O. For our surfaces, selectivity in AS-ALD can be attributed to blocking of active sites (silanols) and/or changing the reaction adsorption/desorption pathway. The effect of TDMAMS on the concentration of free surface silanols was further investigated using HS-LEIS. Finally, for our system, selectivity relies on the stability of adsorbed TDMAMS. To better understand this stability, we investigated the ALD of ZnO onto Si\SiO_2_\TDMAMS that had been deliberately damaged by heating in the air or under N_2_ at 330 °C.

Selectivity in AS-ALD can be represented and displayed in different ways. Selectivity is commonly represented as a selectivity window, which is the number of delayed cycles compared to growth on an uninhibited/unmodified substrate and/or a selectivity value between 0 and 1, which correspond to no selectivity and perfect selectivity, respectively [4,22]. The selectivity window can usually be determined from a plot of thicknesses versus the ALD cycle number. The selectivity value is based on a definition of chemical selectivity, which is derived from the underlying thermodynamics and first order kinetics of the desired and undesired surface reactions. The most common definition the selectivity, S, is the difference between the thicknesses of the films on the unmodified/unpassivated, $\theta_{1}$, and modified, $\theta_{2}$, substrates divided by the sum of those thicknesses (see Equation (1)) [4,22,23].

(1) $$S=\frac{\theta_{1}-\theta_{2}}{\theta_{1}+\theta_{2}}$$

where $\theta_{1}$ and $\theta_{2}$ are the amounts of deposited material on the growth and non-growth surfaces, respectively. Depending on the deposition method, θ can be calculated by different methods including scanning electron microscopy (SEM), transmission electron microscopy (TEM), atomic force microscopy (AFM), SE, or barriers/energy differences from computational studies [24]. Higher selectivity is always desirable. Finally, in addition to showing the selectivity as a function of the number of ALD cycles, the normalized growth per cycle was plotted. This representation of the data appears to effectively identify some of the important regimes in film growth and inhibition in AS-ALD.

## 2. Experimental Section

### 2.1. Materials

Silicon wafers, Si(100), were obtained from University Wafer Inc. (South Boston, MA, USA). Prior to functionalization, they were cleaned using a Basic Plasma Cleaner (115 V) from Harrick Plasma (Ithaca, NY, USA). Tris(dimethylamino)methylsilane, TDMAMS, 97% purity, was obtained from Gelest (Morrisville, PA, USA) and used as received. Dimethylzinc (DMZ) was obtained from Strem Chemicals (Newburyport, MA, USA). HPLC grade water was used for ALD.

### 2.2. TDMAMS Deposition on Silicon Wafers

TDMAMS was deposited on silicon wafers at 200 °C in a previously reported, home-built, flow-through, ambient-pressure reactor [14]. Short, 20 and 30 ms, pulses of TDMAMS and water were carried by 60 and 90 s pulses of nitrogen gas, respectively.

### 2.3. Atomic Layer Deposition (ALD) of ZnO on Passivated Silicon (Si\SiO_2_) 

ALD of ZnO was performed with an ALD-150LX™ system from the Kurt J. Lesker Co. (Jefferson Hills, PA, USA) by applying 21 and 15.5 ms pulses of dimethylzinc (DMZ) and water, respectively. The purging time after each pulse was 10 s. The DMZ and water sources were held at 110 °C and 24 °C, respectively, and the substrate was at 200 °C.

### 2.4. Ex Situ Spectroscopic Ellipsometry (SE)

Ex situ SE was performed with an RC2 instrument from the J.A. Woollam Co. (Lincoln, NE, USA) from 200 to 1000 nm at three angles of incidence (65°, 70°, and 75°). The data from these measurements were modeled over the full wavelength range of the instrument to determine the thickness of the native oxide layer on silicon. Thick (7–8 nm) ZnO films were modeled over their (essentially) transparent region from 420–1000 nm using a Sellmeier model. All data modeling was in Woollam’s CompleteEASE software package (ver. 6.61 a). The ex situ SE modeling of thick ZnO reference sample was performed using a three-layer model that accounted for the silicon substrate; the silicon native oxide layer, which, again, was measured prior to deposition and fixed to the value obtained in this measurement; and a (Sellmeier) layer that accounted for the ZnO film.

### 2.5. In Situ Multi-Wavelength Ellipsometry

In situ SE measurements during ALD of ZnO were performed with a four-wavelength FS-1 ellipsometer from Film Sense (Lincoln, NE, USA), and the data were modeled using Film Sense’s software (Ver 2.66W) [8,25,26]. Two approaches were used to model the data. In the first, the optical constants for ZnO were obtained from an ex situ SE analysis of the ZnO film, which yielded values for n at 464, 523, 597, and 638 nm (the wavelengths of the Film Sense ellipsometer) of 2.01, 2.01, 1.99, and 1.99, respectively [27], and k was taken to be zero at these wavelengths. In the second, the ZnO film was modeled with a Sellmeier function. Both approaches produced similar thicknesses. Only the results from the first approach are shown in this work. In the modeling of the in situ data, the optical constants for heated silicon (at 200 °C) were used.

### 2.6. X-ray Photoelectron Spectroscopy (XPS)

XPS spectra were acquired with a Thermo Scientific Model ESCALAB 250Xi XPS instrument equipped with a monochromatic Al K-alpha X-ray source. All XPS spectra were acquired using a focused X-ray spot size of 650 μm at the sample. XPS survey spectra (0–1350 eV) were acquired using an analyzer pass energy of 150 eV with a 1.0 eV step size and a 10 ms dwell time. High-resolution XPS spectra were acquired with an analyzer pass energy of 20 eV, a 0.1 eV step size, and a 50 ms dwell time. Charge neutralization of the samples during analysis was accomplished using a low-energy electron source that is in-lens in the analyzer column plus an auxiliary combination low-energy electron/low-energy argon ion source in the analysis chamber. The base pressure in the analysis chamber was typically 2.0 × 10^−8^ mbar or better prior to XPS data acquisition. During the XPS data acquisition, the pressure in the analysis chamber was typically 2.0 × 10^−7^ mbar because of the low-energy argon ions leaked into the auxiliary change neutralization source.

### 2.7. High-Sensitivity/Low-Energy Ion Scattering (HS-LEIS)

Ion scattering experiments were performed with a Qtac100 (IonTof GmbH, Münster, Germany) instrument. Small (ca. 100 μm) diameter beams of He^+^ (3.0 keV) were scanned over selected 2 × 2 mm^2^ areas. Surface charging of the samples was compensated with an electron flood gun. During the transport of the samples to the LEIS, they will pick up some (organic) contamination. Therefore, before the LEIS analyses, they were cleaned with atomic oxygen (50 min). Since this may form some ZnO_2_, Zn(IV), this peroxide is converted to the regular oxide (ZnO, Zn(II)) by annealing at 270 °C. All results reported herein are based on three measurements at two different spots.

## 3. Results and Discussion

**Figure 2:**
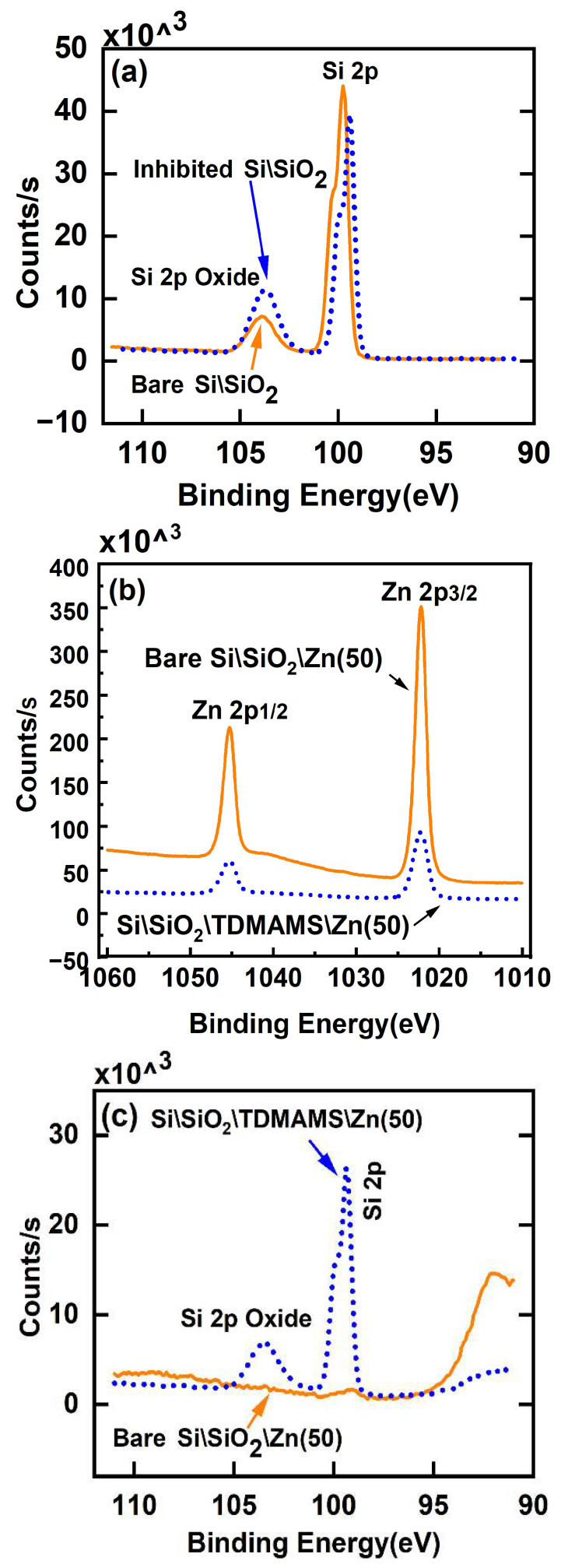
XPS Si 2p narrow scans of (**a**) bare Si\SiO_2_ and Si\SiO_2_\TDMAMS (inhibited Si\SiO_2_), (**b**) Zn 2p peaks of Si\SiO_2_ and Si\SiO_2_\TDMAMS after 50 ALD cycles of DMZ and water, and (**c**) Si 2p signals from the samples in (**b**). In these panels, the solid orange lines correspond to bare Si\SiO_2_, and the dotted blue lines correspond to Si\SiO_2_\TDMAMS.

Passivation of native-oxide-terminated silicon (Si\SiO_2_) with TDMAMS (Si\SiO_2_\TDMAMS) was confirmed by spectroscopic ellipsometry (SE), X-ray photoelectron spectroscopy (XPS), and contact angle goniometry. For the SE, a simple, two-layer model accounted for the silicon substrate and the native oxide layer and was used to obtain the thickness of the native oxide layer on a plasma-cleaned silicon wafer. This same model was then used to find the combined thickness of the native oxide and TDMAMS layers. This approximation should be very good because these layers are very thin and chemically similar [28]. The thickness of the SiO_2_ layer from the unpassivated surface was subtracted from the combined thickness of the SiO_2_ and TDMAMS layers on the passivated surface to yield the thickness of the TDMAMS film, which was 0.54 ± 0.01 nm. The XPS Si 2p narrow scans of clean, bare Si\SiO_2_ before and after TDMAMS passivation are shown in Figure 2a. These narrow scans show the expected bulk Si and oxide Si 2p peaks, where evidence of the underlying spin-orbit splitting is present in the bulk Si 2p peaks [29]. Consistent with the deposition of a thin film of TDMAMS on the native oxide, the ratio of the oxide Si 2p peak to the bulk Si 2p peak increases after TDMAMS deposition. Contact angle goniometry of bare Si\SiO_2_ and Si\SiO_2_\TDMAMS showed that, as expected, the clean Si\SiO_2_ surface was wet with water, e.g., a typical water contact angle was 8°, while, after reaction of Si\SiO_2_ with TDMAMS, the advanced water contact angle (average of three different surfaces) increased dramatically to 81.3 ± 1.5°. It is remarkable that such a thin film/layer of hydrocarbon can have such a profound influence on the wetting of a surface.

**Figure 3:**
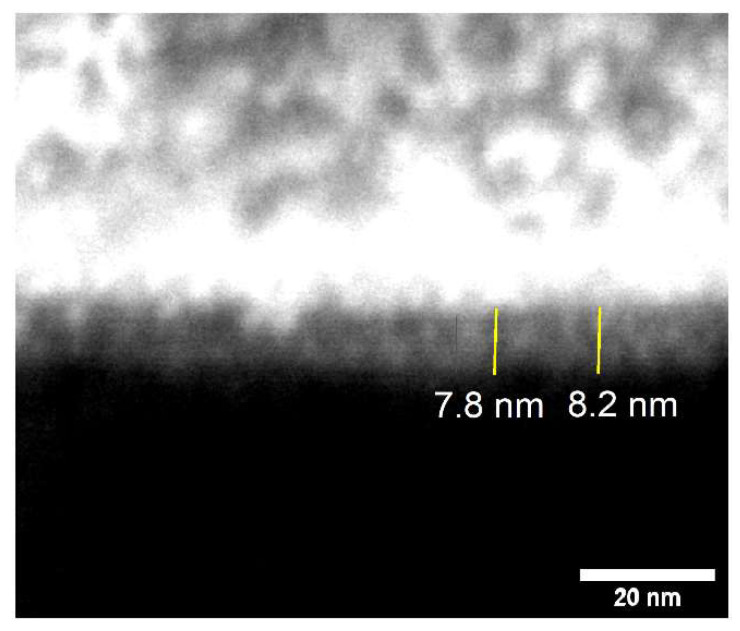
SEM image of a layer of ZnO deposited on a bare Si\SiO_2_ substrate via 50 ALD cycles of DMZ and water. The bright region on top of the image is a protective, conductive Au-Pd\Pt coating. The contrast of this image was enhanced using the ImageJ software package (Java 1.8.0_172) [38].

To test their ability to resist ALD, clean, bare Si\SiO_2_ and Si\SiO_2_\TDMAMS underwent 50 ALD cycles of dimethylzinc (DMZ) and water under conditions that are expected to grow ZnO. XPS shows good evidence for ZnO growth on the unpassivated surface and less growth on the passivated one. For example, after 50 ALD cycles to deposit ZnO, the Zn 2p signals from bare Si\SiO_2_ are noticeably higher than those from Si\SiO_2_\TDMAMS (see Figure 2b). In addition, the Si 2p peaks from the Si\SiO_2_ surface are essentially gone, which is consistent with it being covered with a moderately thick film of ZnO, while the Si 2p peaks from Si\SiO_2_\TDMAMS are still pronounced, suggesting inhibition of ZnO growth (see Figure 2c). The thickness of the ZnO film on uninhibited Si\SiO_2_ was measured by SEM (see Figure 3) and ex situ SE (see Experimental Section) to be 8.0 nm (average of two measurements) and 8.39 nm, respectively. These values are in reasonable agreement.

**Figure 4:**
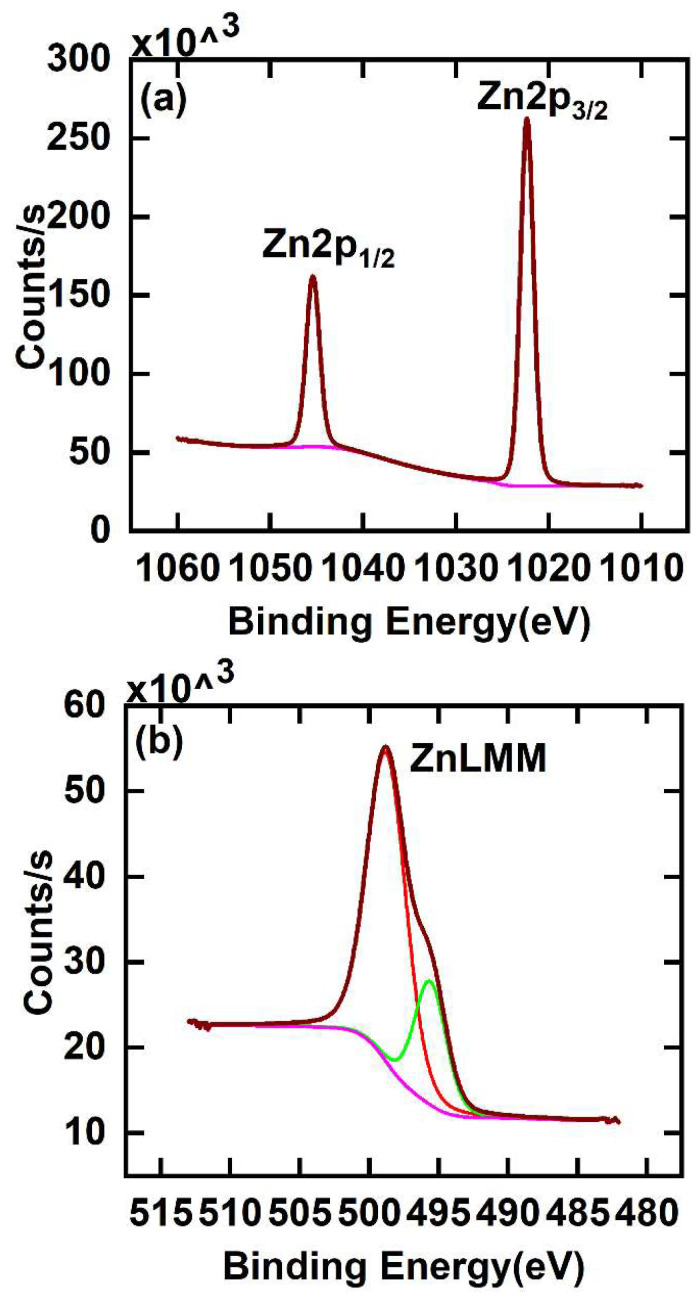
XPS of ZnO deposited on a bare Si\SiO_2_ substrate via 50 ALD cycles of DMZ and water. (**a**) Narrow scan of the Zn 2p region and (**b**) the Zn LMM Auger signal. These signals were fit with LA functions in CasaXPS. These peak fits were only performed to find the positions of the maxima of the Zn 2p_3/2_ and Zn LMM signals from which we obtained the modified Auger parameter. We do not claim that these fits have any chemical significance.

While it is often possible to determine the chemical states of elements by XPS, the Zn(0) and Zn(II) 2p_3/2_ peaks at 1021.4 and 1021.7 eV, respectively, are not widely spaced in energy, which makes chemical state speciation of Zn(0) or Zn(II) from this peak challenging [30]. Accordingly, to confirm the chemical state of zinc in this film, we calculated its modified Auger parameter. The Auger parameter was first introduced by Wagner as the kinetic energy (KE) of a specified photoemission peak subtracted from the KE of a specified Auger peak [31,32]. In many cases, it is both more sensitive to the oxidation states of elements and occurs over a wider range of energies than the positions and differences of corresponding photoemission signals. Auger peaks are often more sensitive to sample chemistry than photoemission peaks because they involve higher energy electrons, i.e., electrons closer in energy to those used for chemical bonding. Gaarenstroom and Winogard added the photon energy to the Auger parameter, which made it independent of the X-ray energy and removed the possibility of it returning negative values [33]. Their form of the Auger parameter is referred to as the modified Auger parameter. Because the modified Auger parameter is the difference between two signals, it is not generally affected by sample charging. To calculate the modified Auger parameter for Zn in our film, we found the positions of the Zn 2p_3/2_ and Zn LMM signals by fitting them in CasaXPS [34,35] with asymmetric Lorentzian (LA) functions (see Figure 4) [35,36]. The modified Auger parameter was then obtained from Equation (2):

Modified Auger parameter (eV) = BE of Zn 2p_3/2_ + KE of Zn LMM (2)

where ‘BE’ stands for ‘binding energy’. The modified Auger parameter we obtained for the zinc in our film is 2010.2 eV, which is the same as that reported elsewhere for ZnO, and very different from that of Zn metal/Zn(0) (2013.8 eV) [37].

**Figure 5:**
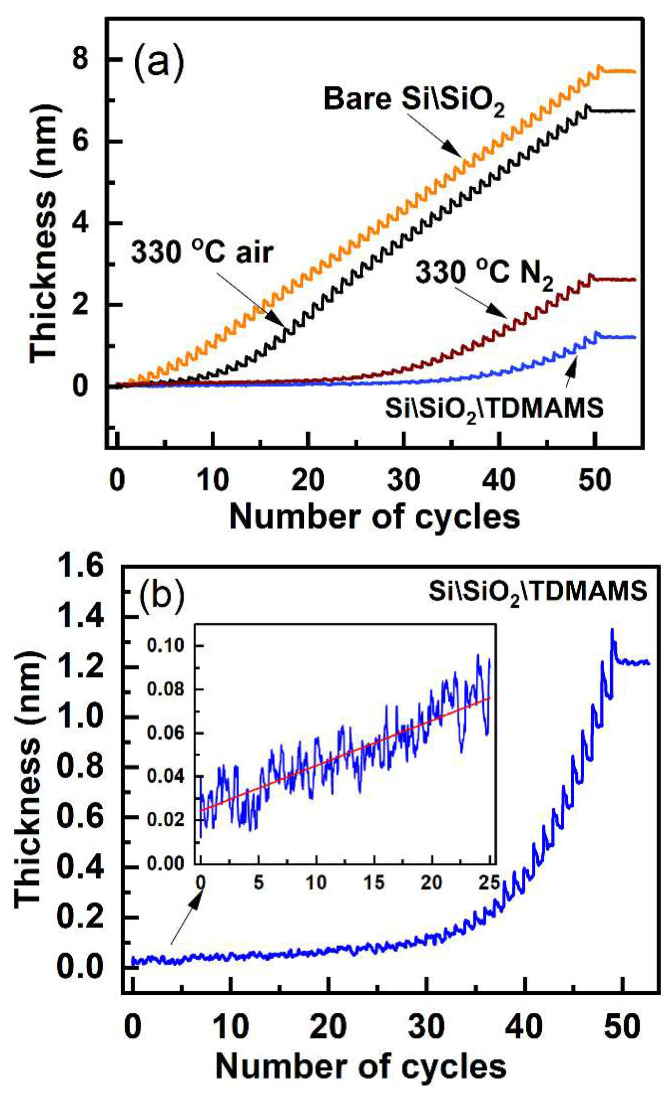
In situ ellipsometry of ZnO grown by thermal ALD of DMZ and H_2_O on (**a**) bare Si\SiO_2_, Si\SiO_2_\TDMAMS, Si\SiO_2_\TDMAMS damaged by heating to 330 °C in N_2_, and Si\SiO_2_\TDMAMS damaged by heating to 330 °C in the air. (**b**) An enlarged view of the results in (**a**) of the in situ ellipsometry of ZnO grown by ALD on Si\SiO_2_\TDMAMS. The inset of this panel shows the ZnO growth for the first 25 cycles of the deposition and a linear fit to these thicknesses.

To assess the inhibitory effects of TDMAMS in ALD, ALD of ZnO was followed by in situ ellipsometry on Si\SiO_2_\TDMAMS and bare Si\SiO_2_. On the unmodified surface (see Figure 5a), there is an induction period of slower growth during the first 5–10 cycles followed by steady, consistent thin-film growth. In contrast, on Si\SiO_2_\TDMAMS, only minimal growth of ZnO takes place during the first 30–35 ALD cycles. Not even 0.1 nm of ZnO has been deposited on Si\SiO_2_\TDMAMS after 30 DMZ-water cycles. These results suggest that TDMAMS interferes with and inhibits ALD of ZnO from DMZ and water. This inhibition is probably a result of the low silanol concentration on the Si\SiO_2_\TDMAMS surface [13], i.e., there are few reactive groups/sites where reaction with DMZ can occur. Furthermore, the relatively few silanols on the Si\SiO_2_\TDMAMS surface may be sterically hindered/inaccessible. Nevertheless, an enlarged view of the in situ ellipsometry of the ALD of ZnO on Si\SiO_2_\TDMAMS suggests very slow, linear growth of ZnO for the first ca. 25 ALD cycles (Figure 5b). In other words, ZnO is being slowly deposited on Si\SiO_2_\TDMAMS. After ca. 30 ALD cycles, ZnO deposition takes place in a more noticeable way. The results in Figure 5 showcase the value of in situ ellipsometry in ALD.

Because many ALD processes take place at higher temperatures (100–400 °C), it is important that inhibited surfaces show good thermal stability. Accordingly, Si\SiO_2_\TDMAMS was deliberately stressed/damaged by heating to 330 °C for 20 min in an inert atmosphere (N_2_) or in the air prior to ALD of ZnO. Of course, exposure to the air under these conditions is expected to be more damaging. The results of ZnO growth on these surfaces are also shown in Figure 5a so that they can be directly compared to those from bare Si\SiO_2_ and undamaged Si\SiO_2_\TDMAMS. Figure 5a shows that the TDMAMS-terminated surface that was heated in nitrogen still has significant inhibitory power, although there is some suggestion that the surface has been damaged. ALD occurs somewhat earlier on this surface than on undamaged Si\SiO_2_\TDMAMS. In contrast, the Si\SiO_2_\TDMAMS surface heated in the air appears to have been substantially damaged. While ALD on this surface is initially slower than on bare Si\SiO_2_, it then takes place quite readily. That is, this ALD shows (i) an induction period with a low but increasing growth rate for ZnO; (ii) a region of rapid/accelerated film growth, which suggests the presence of islands on this surface; and finally, (iii) the same steady-state growth that was observed on the unsilanized surface [22].

**Figure 6:**
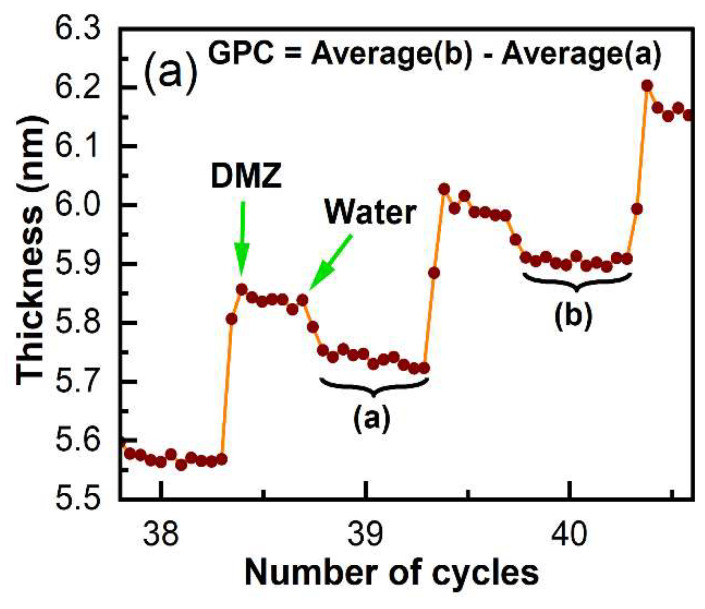

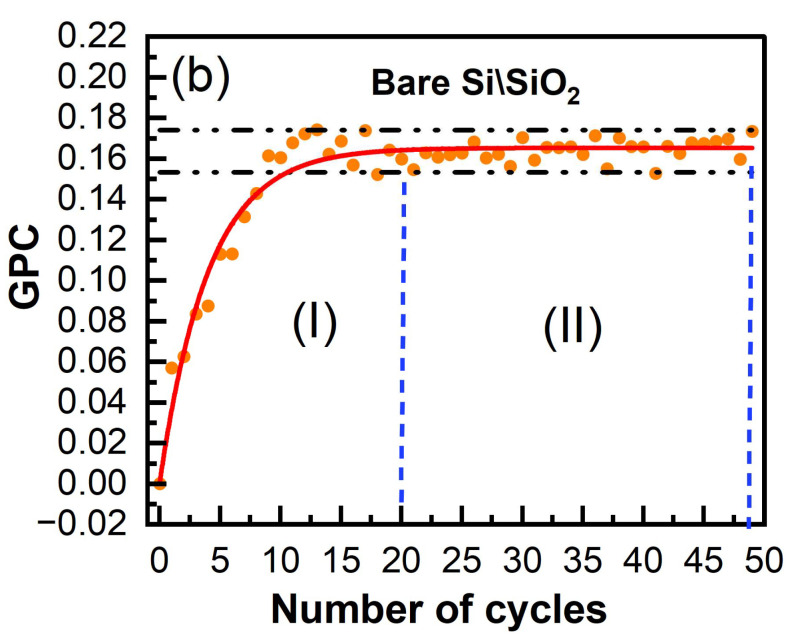
(**a**) An enlarged view of a portion of the in situ ellipsometry results in Figure 5a for the ALD of DMZ and H_2_O on bare Si\SiO_2_ showing an example of the determination/calculation of the growth per cycle (GPC). (**b**) The GPC values for the ALD of DMZ and H_2_O on bare Si\SiO_2_. The red line through the data is a guide for the eye. The parameter 'GPC∞' is defined as the average GPC at and beyond 20 cycles where growth is nearly constant.

**Figure 7:**
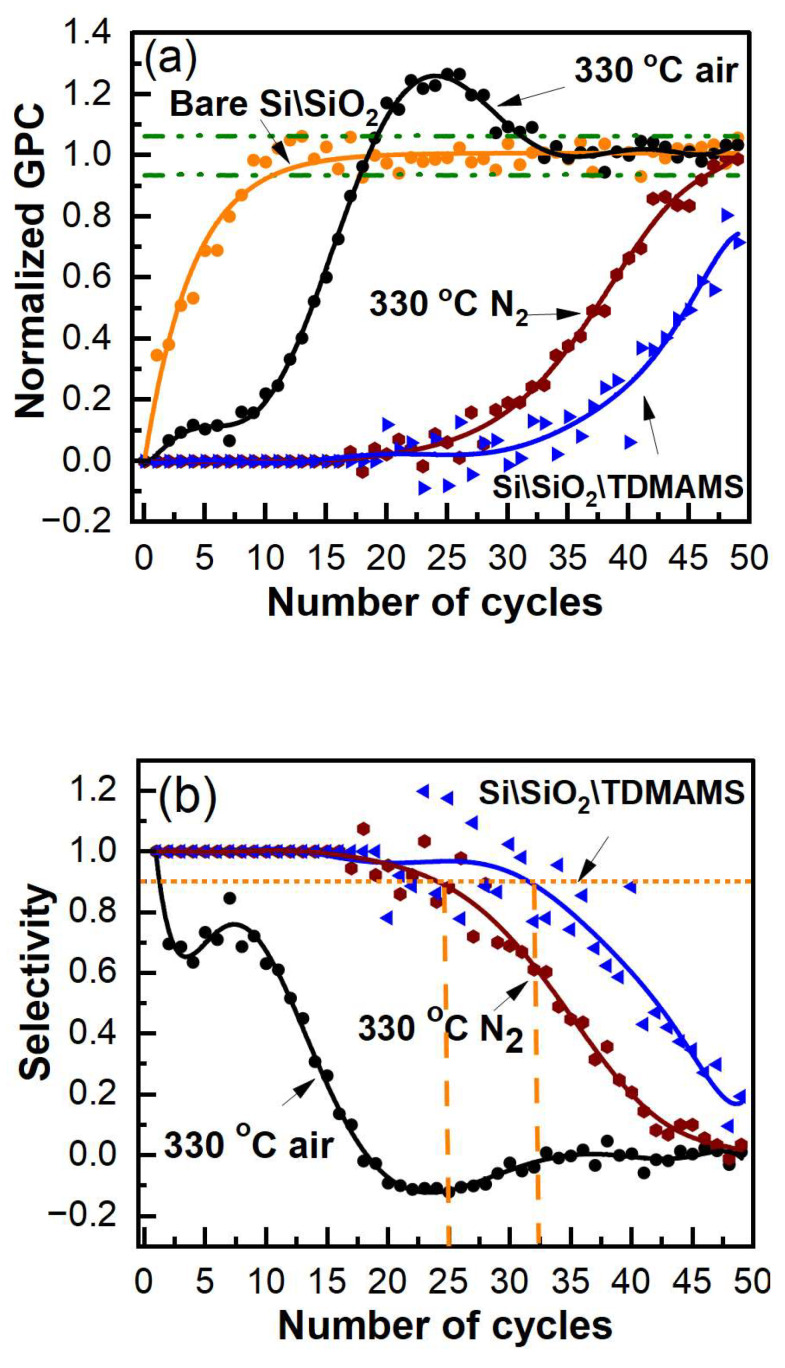
(**a**) Normalized growth per cycle and (**b**) selectivities as a function of the number of ALD cycles for the ZnO ALD growth on bare Si\SiO_2_, Si\SiO_2_\TDMAMS, and Si\SiO_2_\TDMAMS heated to 330 °C in N_2_ or the air in Figure 5a.

The growth per cycle (GPC) is an important figure of merit for ALD. Figure 6a shows a representative example of how we calculated the GPC from our in situ ellipsometry results, and Figure 6b shows the GPCs for all the cycles in the deposition of DMZ and H_2_O on bare Si\SiO_2_ in Figure 5a. As shown in Figure 6b, the GPC for the ALD of ZnO on bare Si\SiO_2_ plateaus after 20 cycles (and maybe after 15). We define the parameter 'GPC∞' for our deposition as the average GPC at and beyond 20 cycles in Figure 6b. All the results in Figure 5a were then normalized to ${GPC}_{\infty }$ to yield the normalized GPCs shown in Figure 7a. This representation of the information appears to facilitate its interpretation. First, since the normalized GPCs for bare Si\SiO_2_ in Figure 7a were obtained from a parameter derived from the same set of data, they do not fundamentally change, except in scale. The normalized GPCs for Si\SiO_2_\TDMAMS that was stressed/damaged by heating to 330 °C for 20 min in the air are more interesting. They show (i) an induction period for the first ca. 18 cycles, (ii) a region of accelerated growth up to ca. cycle 32 that is indicative of island growth where the substrate surface area is higher than after island coalescence (this type of growth is commonly observed in substrate-inhibited ALD [22]), and (iii) a normalized GPC value for the deposition approximately equal to ${GPC}_{\infty }$ thereafter, which suggests that island coalescence has taken place at this point. Finally, the GPC of the Si\SiO_2_\TDMAMS surface that was stressed/damaged by heating to 330 °C in N_2_ reaches ${GPC}_{\infty }$ after ca. 47 cycles, while the unstressed Si\SiO_2_\TDMAMS surface has still not reached ${GPC}_{\infty }$ at 50 ALD cycles.

The traditional selectivities [3] for the depositions shown in Figure 5a are presented in Figure 7b, where the threshold for selectivity (0.9) that is commonly used for comparison purposes is indicated [22]. As expected, the selectivity is highest (32 cycles) for the intact TDMAMS surface. It is moderately diminished for the surface heated to 330 °C in N_2_ (25 cycles), and strongly diminished for the surface heated to 330 °C in the air (2 cycles). Both intact Si\SiO_2_\TDMAMS and Si\SiO_2_\TDMAMS stressed under N_2_ show good selectivity.

**Figure 8:**
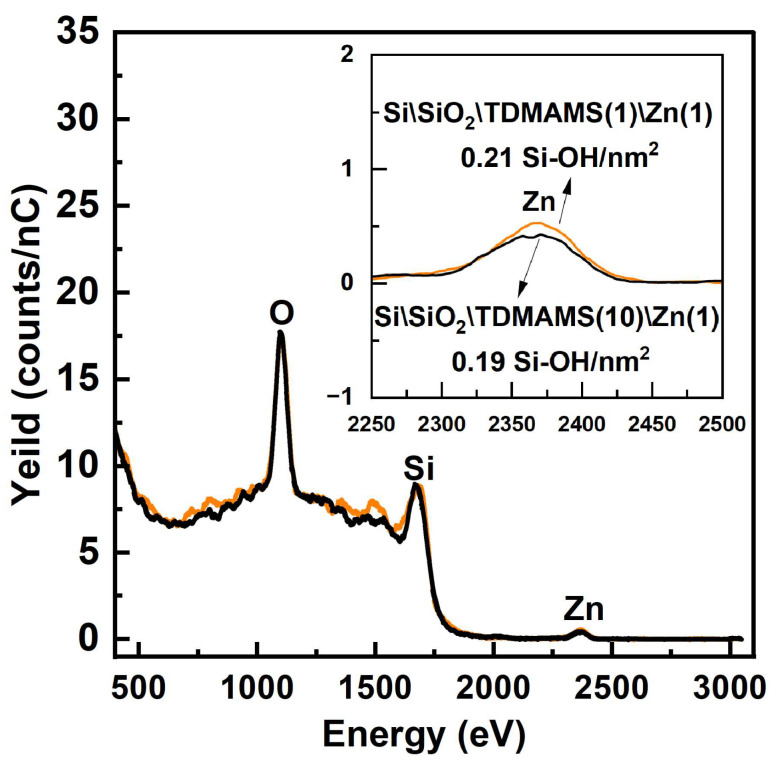
LEIS spectra of Si\SiO_2_ passivated with one pulse of TDMAMS and then reacted with DMZ (Si\SiO_2_\TDMAMS(1)\Zn(1)) and Si\SiO_2_ passivated with ten pulses of TDMAMS and then reacted with DMZ (Si\SiO_2_\TDMAMS(10)\Zn(1)). The inset of this figure is an enlargement of the Zn region of the spectra shown in the main panel.

Our previous work had shown that ALD between TDMAMS and water is not possible [13]. That is, previously, multiple cycles of TDMAMS and H_2_O did not increase the film thickness beyond what was obtained in the first deposition of TDMAMS. We follow up on these results by testing whether there is a difference in the number of silanols remaining on Si\SiO_2_ after exposing it to one or ten pulses of TDMAMS, where no water was applied until after the end of these pulses. After tagging with Zn, HS-LEIS shows little difference between the number of silanols, i.e., Zn atoms (see Figure 8), on these surfaces. These results (ca. 0.20 silanols/nm^2^) are in reasonable agreement with our previous LEIS results (0.31 silanols/nm^2^). Thus, one short (30 ms) pulse of TDMAMS, or even multiple pulses of this reagent, passivate Si\SiO_2_ to a significant extent.

## 4. Conclusions

TDMAMS deposition on Si\SiO_2_ and AS-ALD of ZnO on this surface were confirmed by various surface analytical techniques, including XPS, in situ and ex situ ellipsometry, wetting, and HS-LEIS. This characterization showed that TDMAMS deposits onto silicon surfaces in a self-limiting way and that it dramatically reduces the number of free silanols on the surface. TDMAMS-passivated Si\SiO_2_ delayed ALD of ZnO for 32 cycles at a selectivity value of 0.9. To test its stability, Si\SiO_2_\TDMAMS was stressed/heated to 330 °C in the air and in nitrogen. The surface was not considerably damaged by heating in N_2_ but was seriously damaged in the air. The surface damaged in the air showed island growth of ZnO at early times. Plots of the normalized GPC facilitated the interpretation of the ALD of ZnO on TDMAMS-terminated surfaces. Overall, TDMAMS is reasonably effective at preventing ALD of ZnO. It is, potentially, an industrial-friendly, small, surface-reactive molecule that may be useful for AS-ALD.

## Data Availability

The data used in this study will be made available upon reasonable request.

## References

[1] Puurunen R.L. (2014). A Short History of Atomic Layer Deposition: Tuomo Suntola’s Atomic Layer Epitaxy. Chem. Vap. Depos..

[2] Kääriäinen T., Cameron D., Kääriäinen M.-L., Sherman A. (2013). Atomic Layer Deposition: Principles, Characteristics, and Nanotechnology Applications.

[3] Mackus A.J.M., Merkx M.J.M., Kessels W.M.M. (2019). From the Bottom-Up: Toward Area-Selective Atomic Layer Deposition with High Selectivity. Chem. Mater..

[4] Parsons G.N., Clark R.D. (2020). Area-Selective Deposition: Fundamentals, Applications, and Future Outlook. Chem. Mater..

[5] Mameli A., Karasulu B., Verheijen M.A., Mackus A.J.M., Kessels W.M.M., Roozeboom F. (2017). (Invited) Area-Selective Atomic Layer Deposition: Role of Surface Chemistry. ECS Trans..

[6] Chen R., Bent S.F. (2006). Chemistry for Positive Pattern Transfer Using Area-Selective Atomic Layer Deposition. Adv. Mater..

[7] Mackus A.J.M. (2018). Approaches and Opportunities for Area-Selective Atomic Layer Deposition. 2018 International Symposium on VLSI Technology, Systems and Application (VLSI-TSA).

[8] Mameli A., Karasulu B., Verheijen M.A., Barcones B., Macco B., Mackus A.J.M., Kessels W.M.M.E., Roozeboom F. (2019). Area-Selective Atomic Layer Deposition of ZnO by Area Activation Using Electron Beam-Induced Deposition. Chem. Mater..

[9] Weber M.J., Mackus A.J.M., Verheijen M.A., van der Marel C., Kessels W.M.M. (2012). Supported Core/Shell Bimetallic Nanoparticles Synthesis by Atomic Layer Deposition. Chem. Mater..

[10] Mackus A.J.M., Mulders J.J.L., Van De Sanden M.C.M., Kessels W.M.M. (2010). Local Deposition of High-Purity Pt Nanostructures by Combining Electron Beam Induced Deposition and Atomic Layer Deposition.

[11] Chang C.-W., Hsu H.-H., Hsu C.-S., Chen J.-T. (2021). Achieving Area-Selective Atomic Layer Deposition with Fluorinated Self-Assembled Monolayers. J. Mater. Chem. C.

[12] Xu W., Haeve M.G.N., Lemaire P.C., Sharma K., Hausmann D.M., Agarwal S. (2022). Functionalization of the SiO_2_ Surface with Aminosilanes to Enable Area-Selective Atomic Layer Deposition of Al_2_O_3_. Langmuir.

[13] Moeini B., Joshua W.P., Avval T.G., Jacobsen C., Brongersma H.H., Prusa S., Bábík P., Vaníčková E., Argyle M.D., Strohmeier B.R. (2023). Controlling the Surface Silanol Density in Capillary Columns and Planar Silicon via the Self-Limiting Gas-Phase Deposition of Tris(dimethylamino)Methylsilane, and Quantification of Surface Silanols after Silanization by Low Energy Ion Scattering.

[14] Patel D.I., Major G.H., Jacobsen C., Shah D., Strohmeier B.R., Shollenberger D., Bell D.S., Argyle M.D., Linford M.R. (2022). Flow-Through Atmospheric Pressure-Atomic Layer Deposition Reactor for Thin-Film Deposition in Capillary Columns. Anal. Chem..

[15] Tonezzer M., Dang T.T.L., Bazzanella N., Nguyen V.H., Iannotta S. (2015). Comparative Gas-Sensing Performance of 1D and 2D ZnO Nanostructures. Sens. Actuators B Chem..

[16] Ding M., Guo Z., Zhou L., Fang X., Zhang L., Zeng L., Xie L., Zhao H. (2018). One-Dimensional Zinc Oxide Nanomaterials for Application in High-Performance Advanced Optoelectronic Devices. Crystals.

[17] Sushma C., Girish Kumar S. (2017). Advancements in the Zinc Oxide Nanomaterials for Efficient Photocatalysis. Chem. Pap..

[18] Ellmer K., Klein A., Rech B. (2007). Transparent Conductive Zinc Oxide: Basics and Applications in Thin Film Solar Cells.

[19] Hirao T., Furuta M., Furuta H., Matsuda T., Hiramatsu T., Hokari H., Yoshida M., Ishii H., Kakegawa M. (2007). Novel Top-gate Zinc Oxide Thin-film Transistors (ZnO TFTs) for AMLCDs. J. Soc. Inf. Disp..

[20] Guziewicz E., Krajewski T.A., Przezdziecka E., Korona K.P., Czechowski N., Klopotowski L., Terziyska P. (2020). Zinc Oxide Grown by Atomic Layer Deposition: From Heavily n-Type to p-Type Material. Phys. Status Solidi.

[21] Chen M.-C., Chang T.-C., Tsai C.-T., Huang S.-Y., Chen S.-C., Hu C.-W., Sze S.M., Tsai M.-J. (2010). Influence of Electrode Material on the Resistive Memory Switching Property of Indium Gallium Zinc Oxide Thin Films. Appl. Phys. Lett..

[22] Parsons G.N. (2019). Functional Model for Analysis of ALD Nucleation and Quantification of Area-Selective Deposition. J. Vac. Sci. Technol. A.

[23] Gladfelter W.L. (1993). Selective Metalization by Chemical Vapor Deposition. Chem. Mater..

[24] Longo R.C., McDonnell S., Dick D., Wallace R.M., Chabal Y.J., Owen J.H.G., Ballard J.B., Randall J.N., Cho K. (2014). Selectivity of Metal Oxide Atomic Layer Deposition on Hydrogen Terminated and Oxidized Si(001)-(2 × 1) Surface. J. Vac. Sci. Technol. B.

[25] Hilfiker J.N. (2011). 5-In Situ Spectroscopic Ellipsometry (SE) for Characterization of Thin Film Growth. Woodhead Publishing Series in Electronic and Optical Materials.

[26] Maynard H.L., Layadi N., Lee J.T.C. (1998). Plasma Etching of Submicron Devices: In Situ Monitoring and Control by Multi-Wavelength Ellipsometry. Thin Solid Film..

[27] Fang L., Li H., Ma X., Song Q., Chen R. (2020). Optical Properties of Ultrathin ZnO Films Fabricated by Atomic Layer Deposition. Appl. Surf. Sci..

[28] Tompkins H.G. (2006). A User’s Guide to Ellipsometry.

[29] Jensen D.S., Kanyal S.S., Madaan N., Vail M.A., Dadson A.E., Engelhard M.H., Linford M.R. (2013). Silicon (100)/SiO_2_ by XPS. Surf. Sci. Spectra.

[30] Biesinger M.C., Lau L.W.M., Gerson A.R., Smart R.S.C. (2010). Resolving Surface Chemical States in XPS Analysis of First Row Transition Metals, Oxides and Hydroxides: Sc, Ti, V, Cu and Zn. Appl. Surf. Sci..

[31] Wagner C.D. (1975). Chemical Shifts of Auger Lines, and the Auger Parameter. Faraday Discuss. Chem. Soc..

[32] Wagner C.D., Joshi A. (1988). The Auger Parameter, Its Utility and Advantages: A Review. J. Electron Spectros. Relat. Phenom..

[33] Gaarenstroom S.W., Winograd N. (2008). Initial and Final State Effects in the ESCA Spectra of Cadmium and Silver Oxides. J. Chem. Phys..

[34] Ortega S., Halicek M., Fabelo H., Callico G.M., Fei B. (2020). Hyperspectral and Multispectral Imaging in Digital and Computational Pathology: A Systematic Review [Invited]. Biomed. Opt. Express.

[35] Fairley N., Fernandez V., Richard-Plouet M., Guillot-Deudon C., Walton J., Smith E., Flahaut D., Greiner M., Biesinger M., Tougaard S. (2021). Systematic and Collaborative Approach to Problem Solving Using X-Ray Photoelectron Spectroscopy. Appl. Surf. Sci. Adv..

[36] Hesse R., Streubel P., Szargan R. (2007). Product or Sum: Comparative Tests of Voigt, and Product or Sum of Gaussian and Lorentzian Functions in the Fitting of Synthetic Voigt-Based X-Ray Photoelectron Spectra. Surf. Interface Anal..

[37] Dake L.S., Baer D.R., Zachara J.M. (1989). Auger Parameter Measurements of Zinc Compounds Relevant to Zinc Transport in the Environment. Surf. Interface Anal..

[38] Collins T.J. (2007). ImageJ for Microscopy. Biotechniques.

